# Editorial

**DOI:** 10.1002/elsc.202170023

**Published:** 2021-03-02

**Authors:** Juliane Steingroewer

**Affiliations:** ^1^ Institute for Natural Product Technology TU Dresden

This special issue is dedicated to Prof. Dr. rer. nat. Thomas Bley in honor of his 70th birthday. Professor Bley is a remarkable person and a commendable scientist. He has rendered outstanding services not only to bioprocess engineering in Germany, but also across national borders.

Prof. Bley was born in Großenhain in 1951 and studied mathematics at the TU Dresden from 1971 to 1975, before he received his doctorate in mathematical biology from the Academy of Sciences of the GDR in 1981. He completed his habilitation in the field of biotechnology at the University of Leipzig in 1990 and thereupon, he was granted the teaching license in this scientific field in 1995. In 1996, Prof. Bley was appointed to the Chair of Biochemical Engineering at the TU Dresden, which he headed until his retirement in 2017. To this day, he is as senior professor closely associated with the institute and the chair and is held in high esteem by staff, doctoral candidates and students alike.

During his teaching career, he led 250 graduate engineers and 45 doctoral students to successful graduation. The “Diplomingenieur” degree is very important to Professor Bley. Therefore, he successfully committed himself to maintaining this degree at the Faculty of Mechanical Engineering of the TU Dresden. Many of the engineers he educated work in leading positions today, in industry and science, nationally and internationally. Professor Bley is particularly proud that one of his first doctoral students and preferred candidate, Prof. Thomas Walther, was appointed his successor to the Chair of Biochemical Engineering at TU Dresden in 2017.

With more than 170 publications, which focus on the mathematical description of complex cell‐cell and cell‐reactor interactions, Professor Bley looks back on a very successful scientific career. In addition to his scientific work, he also created important impulses nationwide through his membership in various expert committees. Since 2002, he is an elected member of the Saxon Academy of Sciences and Humanities and the German Academy of Science and Engineering. In the years 2008–2013, he was an elected member of the DFG Review Board for Process Engineering and Technical Chemistry. In addition, he committed very actively in the DECHEMA for many years, among others as chair of the Bioprocessing Working Group from 2009 to 2014, and from 2013 to 2016 as a member of the Board of the DECHEMA Section on Biotechnology. In 2018, Professor Bley was therefore awarded the DECHEMA Medal. The DECHEMA awards this medal for outstanding commitment in realizing its objectives and for outstanding achievements in the field of chemical engineering and biotechnology. With this award, the DECHEMA recognized his commitment to the bioprocessing community: “*Thomas Bley has actively brought together industry and academia, young and experienced scientists and ‐ in the course of Germany's reunification ‐ experts from Eastern and Western Germany. In addition, he provided scientific impetus in numerous ways*”.

Dear Thomas, at this point, I would like to take the opportunity to thank you on behalf of all the staff of the Chair of Bioprocess Engineering for the work that you have done! The work under your leadership was a valuable enrichment for me and the other colleagues in every respect, both professionally and personally. Due to the wide range of research topics at the chair and the fruitful working environment, there was always the opportunity to dedicate oneself to new scientific areas. The research groups, which you have initiated, including plant cell and algae technology, smart labs systems, and enzyme technology, are continuing to work sustainably and they provide the ground for successful scientific careers in the future, for which your former colleagues and I are most grateful.

You accompanied me on my scientific, but also personal career path as a university professor, doctoral advisor and supervisor for many years. I would like to thank you for your trust in my scientific work and the many opportunities, as well as for the fact that you always had a diplomatically advice and continuously supported my personal scientific career.

I congratulate you on your 70th birthday and wish you all the best, many wonderful hours in your garden and with your grandchildren and above all: health.

